# Corneal Confocal Microscopy Detects Small Fibre Neuropathy in Patients with Upper Gastrointestinal Cancer and Nerve Regeneration in Chemotherapy Induced Peripheral Neuropathy

**DOI:** 10.1371/journal.pone.0139394

**Published:** 2015-10-02

**Authors:** Maryam Ferdousi, Shazli Azmi, Ioannis Nikolaos Petropoulos, Hassan Fadavi, Georgios Ponirakis, Andrew Marshall, Mitra Tavakoli, Imaan Malik, Wasat Mansoor, Rayaz Ahmed Malik

**Affiliations:** 1 Institute of Human Development, Centre for Endocrinology & Diabetes, Faculty of Medical and Human Sciences, University of Manchester and NIHR/Wellcome Trust Clinical Research Facility, Manchester, United Kingdom; 2 Department of Clinical Neurophysiology, Manchester Royal Infirmary, Central Manchester University Hospitals NHS Foundation Trust, Manchester Academic Health Science Centre, Manchester, United Kingdom; 3 Weill Cornell Medical College Qatar, Division of Research, Qatar Foundation, Education City, Doha, Qatar; 4 The Christie NHS Foundation Trust, Manchester, United Kingdom; Hirosaki University Graduate School of Medicine, JAPAN

## Abstract

There are multiple neurological complications of cancer and its treatment. This study assessed the utility of the novel non-invasive ophthalmic technique of corneal confocal microscopy in identifying neuropathy in patients with upper gastrointestinal cancer before and after platinum based chemotherapy. In this study, 21 subjects with upper gastrointestinal (oesophageal or gastric) cancer and 21 healthy control subjects underwent assessment of neuropathy using the neuropathy disability score, quantitative sensory testing for vibration perception threshold, warm and cold sensation thresholds, cold and heat induced pain thresholds, nerve conduction studies and corneal confocal microscopy. Patients with gastro-oesophageal cancer had higher heat induced pain (P = 0.04) and warm sensation (P = 0.03) thresholds with a significantly reduced sural sensory (P<0.01) and peroneal motor (P<0.01) nerve conduction velocity, corneal nerve fibre density (CNFD), nerve branch density (CNBD) and nerve fibre length (CNFL) (P<0.0001). Furthermore, CNFD correlated significantly with the time from presentation with symptoms to commencing chemotherapy (r = -0.54, P = 0.02), and CNFL (r = -0.8, P<0.0001) and CNBD (r = 0.63, P = 0.003) were related to the severity of lymph node involvement. After the 3^rd^ cycle of chemotherapy, there was no change in any measure of neuropathy, except for a significant increase in CNFL (P = 0.003). Corneal confocal microscopy detects a small fibre neuropathy in this cohort of patients with upper gastrointestinal cancer, which was related to disease severity. Furthermore, the increase in CNFL after the chemotherapy may indicate nerve regeneration.

## Introduction

Neurological complications of cancer include direct tumour compression or infiltration, paraneoplastic neurological syndromes (PNS), metabolic and nutritional deficiencies and chemotherapy induced peripheral neuropathy (CIPN) [1]. CIPN has a detrimental impact on the patients' quality of life [2], has limited treatment options [3] and may lead to chemotherapy dose reduction or discontinuation [4]. Cisplatin and Oxaliplatin are known to cause problematic dose limiting peripheral neuropathies, particularly in patients receiving standard of care treatments for gastro-oesophageal cancers [5].

The aetiology of CIPN remains to be determined [1]. A recent study has shown that paclitaxel-induced neuropathy has a heritable component, driven in part by genes involved in axon outgrowth [6]. Early objective detection of neuropathy is important to enable clinicians to identify patients who may already have neuropathy and who may therefore be more likely to develop CIPN to enable an alteration in chemotherapy doses. Currently physicians record the patient’s subjective history of neurologic symptoms using the Common Terminology Criteria of Clinical Adverse Events (CTCAE) [7]. A number of symptom and deficit based neuropathy focused scales have been proposed for assessing chemotherapy induced neuropathy [8–10]. More accurate diagnostic tests include nerve conduction studies (NCS), quantitative sensory testing (QST) and skin biopsy for measuring the intra epidermal nerve fibre density (IENFD) [11]. However, NCS assesses large fibres only, QST is subjective and skin biopsy is invasive and requires expertise for quantification. Alternatively, nerve morphology can be rapidly assessed using in vivo corneal confocal microscopy (IVCCM), and over the past decade we have pioneered the use of this technique to assess peripheral neuropathy in a range of neuropathies including diabetic neuropathy [12–14], Idiopathic small fibre neuropathy (ISFN) [15], Fabry’s disease [16] and Charcot-Marie-Tooth Neuropathy Type 1 (CMT1A) [17]. Others have deployed it in non-length dependent and autoimmune neuropathies [18, 19]. A recent experimental study has shown a paclitaxel induced dose-dependent loss of small nerve fibres in both the skin and cornea of mice [20]. To date there is one case report of a 75-year-old man with metastatic colorectal carcinoma receiving capecitabine who developed CIPN, and CCM showed increased beading, tortuosity and sprouting of the corneal nerves [21]. We have therefore undertaken a detailed study using CCM in patients with upper GI cancer before and after receiving chemotherapy.

## Methodology

This study was approved by the North Manchester Research Ethics Committee, and adhered to the tenets of the Declaration of Helsinki. Patients were recruited from The Christie Hospital Foundation Trust, Manchester, UK. Informed written consent was obtained from all the participants. 21 patients scheduled to undergo either curative or palliative chemotherapy with Oxaliplatin or Cisplatin containing regimens were examined at baseline. Exclusion criteria were other causes of neuropathy such as diabetes, deficiency of vitamin B_12_ or folate, autoimmune disease and any history of systemic or ocular disease with corneal involvement. Only 13 patients were examined after 3 cycles of chemotherapy due to voluntary withdrawal of consent, disease progression and disability or death.

### Clinical and neurological examination

All study participants including 21 patients and 21 aged matched healthy controls underwent assessment of Body Mass Index (BMI), glycated haemoglobin (HbA1c), serum B_12_ and folate levels. Symptoms were assessed using the Neuropathy Symptom Profile (NSP) and the McGill pain questionnaire pre and post treatment and NCI Common Terminology Criteria for Adverse Events (CTCAE) (Version 4) post treatment. Neurological deficits were evaluated using the simplified neuropathy disability score (NDS). Vibration perception threshold (VPT) was assessed using a Neuroesthesiometer (Horwell, Scientific Laboratory Supplies, Wilford, Nottingham, UK) on the hallux of both feet, and cold and warm sensation threshold (CST & WST); and cold and warm induced pain threshold (CIP & WIP) were assessed with a TSA-II NeuroSensory Analyser (Medoc Ltd., Ramat-Yishai, Israel) on the dorsolateral aspect of the left foot. Sural sensory nerve amplitude (SSNamp), sural sensory nerve conduction velocity (SSNCV), peroneal motor nerve amplitude (PMNamp) and peroneal motor nerve conduction velocity (PMNCV) were assessed using a Dantec “Keypoint” system (Dantec Dynamics Ltd, Bristol, UK).

### Corneal assessment

Corneal sensitivity was measured using non-contact corneal aesthesiometry (NCCA) [22] (Glasgow Caledonian University, Glasgow, Scotland, UK). Corneal imaging was undertaken using a laser CCM (Heidelberg Retinal Tomograph III Rostock Cornea Module HRT III RCM; Heidelberg Engineering GmbH, Heidelberg, Germany) according to our established protocol [23]. Six images (3 from each eye) from the corneal sub-basal nerve plexus, taking into account the depth, contrast and position from the centre of cornea were exported and analysed by a single experienced examiner using purpose-designed software (CCMetrics (MA Dabbah; Imaging Science and Biomedical Engineering, University of Manchester, Manchester, UK)) in a masked fashion [24]. The outcome measures from these 6 images were averaged and used for statistical analysis. Corneal nerve morphological parameters included corneal nerve fibre density (CNFD)—number of main nerve fibres/mm^2^; corneal nerve branch density (CNBD)—number of branch points on the main nerves/mm^2^; and corneal nerve fibre length (CNFL)—total length of nerves mm/mm^2^.

### Statistical analysis

IBM SPSS v19.0 (Chicago, IL, USA) for Windows was used to compute the results. All the data were expressed as Mean ± SD and analysis included descriptive and frequency statistics. Independent sample t tests (Mann-Whitney U test for non-parametric) were used to evaluate between group differences. Paired sample t tests (Wilcoxon for non-parametric) were used to evaluate differences between patients at baseline and follow up. One-way ANOVA with Bonferoni adjustment was used to evaluate between group differences. The association between the corneal nerve morphological parameters and clinical findings was assessed using the Pearson correlation coefficient (Spearman for non-parametric). Pearson’s Chi-square (χ^2^) test of independence and a Fisher’s Exact test were used to evaluate the association between the categorical variables. For all the comparisons, a *P* < 0.05 was considered significant. The data used for statistical analysis in this study can be found at http://dx.doi.org/10.6084/m9.figshare.1449256.

## Results

### Baseline

21 subjects (oesophageal cancer (*n* = 12), gastric cancer (*n* = 6), gastro-oesophageal junction cancer (*n* = 3) with (*n* = 15) and without (*n* = 6) metastases and 21 age and gender matched healthy control subjects were assessed. The clinical and demographic characteristics of patients and healthy controls are summarised in Table 1. There was no significant difference in age, BMI or alcohol and cigarette consumption between groups. HbA1c was significantly lower (P<0.05), B12 (P<0.05) was higher and folate (P<0.05) was lower compared to controls, however all values were within the normal range.

**Table 1:** Clinical and demographic characteristics in patients with upper GI cancer and age matched healthy controls.

Parameters	Control	Patients
**Number (female/male)**	21(2/20)	21(1/20)
**Age (Years)**	60.2±8.7	63.8±10.0
**BMI (kg/m** ^**2**^ **)**	27.4±4.3	27.0±6.4
**Alcohol consumption (units per week)**	6.7±9.2	14.4±15.3
**Smoking (cigarettes per day)**	0.9±3.0	4.5±7.9
**HbA1c (mmol/mol)**	39.7±2.9	34.5±6.1#
**Serum B12 (ng/l)**	245.6±77.4	668.2±383.2#
**Serum Folate (ug/L)**	10.3±5.6	2.7±0.58#
**Anatomy (GAST/OES/GOJ)**	-	(6/12/3)
**Histology (ADENO/SQ)**	-	(15/6)
**Node**	-	1.3±0.9
**Metastases (present/absent)**	-	(15/6)
**Stage**	-	3.5±0.8

All data are presented as Mean ± SD. All symbols represent statistically significant differences

*P<0.01

$P<0.001

#P<0.05

Gastric (GAST), Oesophageal (OES); Gastro oesophageal junction (GOJ); Adenocarcinoma (ADENO); Squamous cell carcinomas (SQ).

Neuropathy findings are summarised in Table 2. The HIP (P = 0.04) and WST (P = 0.03) were significantly higher and SSNCV and PMNCV were significantly (P = 0.01) lower, with no difference in the NDS, VPT, CST, CIP, SNamp, PMNamp or corneal sensation between patients and control subjects. However, CNFD (P<0.0001), CNBD (P<0.0001) and CNFL (P<0.0001) were significantly lower in patients compared to control subjects. Furthermore, CNFD correlated significantly with the time from presentation with symptoms to the time taken to receive the first cycle of chemotherapy (r = -0.54, P = 0.02). There were also highly significant correlations between CNBD (r = -0.8, P<0.0001) and CNFL (r = -0.63, P = 0.003) with the number of lymph nodes (>1cm) involved. There was no significant correlation between corneal nerve morphology and the type or stage of cancer, or the presence of metastases.

**Table 2:** Baseline assessment of neuropathy in patients with upper GI cancer and age matched healthy controls.

Parameters	Control	Patients
**NSP (0–38)**	0.67±1.06	0.83±1.7
**McGill pain index (0–5)**	0.29±0.96	0.12±0.5
**NDS (0–10)**	0.84±1.16	1.39±2.09
**VPT (volts)**	9.91±6.24	13.9±10.8
**CST (**°**C)**	27.99±2	26.8±2.1
**WST (**°**C)**	37.8±2.3	39.6±2.3#
**CIP (**°**C)**	13.01±8.72	10.1±8.1
**HIP (**°**C)**	43.39±10.33	46.8±2.8#
**SSNCV (m/s)**	48.14±3.12	44.1±6.3*
**SSNamp (μA)**	12.39±6.59	9.68±4.6
**PMNCV (m/s)**	46.04±3.62	42.7±3.8*
**PMNamp (mV)**	4.97±2.32	3.8±2.3
**NCCA (mbars)**	0.64±0.36	0.61±0.36
**CNFD (no./mm** ^**2**^ **)**	37.13±6.28	25.34±4.85^$^
**CNBD (no./mm** ^**2**^ **)**	98.43±33.2	50.84±28.38^$^
**CNFL (mm/mm** ^**2**^ **)**	26.82±4.27	18.08±3.62^$^

All data are presented as Mean ± SD. Symbols represent statistically significant differences

*P<0.01

$P<0.001

#P<0.0.

NSP (Neuropathy Symptom Profile), NDS (Neuropathy Disability Score), VPT (Vibration perception threshold), CST (Cold Sensation Threshold), WST (Warm Sensation Threshold), CIP (Cold Induced Pain), HIP (Heat Induced Pain), SSNCV (Sural Sensory Nerve Conduction Velocity), SSNamp (Sural Sensory Nerve Amplitude), PMNCV (Peroneal Motor Nerve Conduction Velocity), PMNamp (Peroneal Motor Nerve Amplitude), NCCA (Non-Contact Corneal Aesthesiometer), CNFD (Corneal Nerve Fibre Density), CNBD (Corneal Nerve Branch Density), CNFL (Corneal Nerve Fibre Length), no. (number).

### Follow up

Of the 13 patients who attended for the follow up, eight received 3 cycles of Oxaliplatin containing regimes [EOX regimes; Oxaliplatin (130mg/m^2^), Epirubicin and Capecitabine] and five received 3 cycles of Cisplatin containing regimes [ECX regimes (*n* = 3); Cisplatin (60 mg/m^2^), Epirubicin and Capecitabine] and [CXH regimes (*n* = 2); Cisplatin (80mg/m^2^), Capecitabine and Herceptin]. Dose intensity for all 13 patients was maintained without any dose reductions or dose delays. 8/13 patients developed grade 1 symptomatic paraesthesia on CTCAE criteria. This was significantly higher in the patients who received Oxaliplatin compared to Cisplatin containing regimens (78% vs. 20%, P = 0.03) (S1 Table). There were no significant difference for any baseline or follow up measure of neuropathy between patients who did and did not develop grade 1 symptomatic paraesthesia on CTCAE criteria (S2 Table).

**Fig 1:**
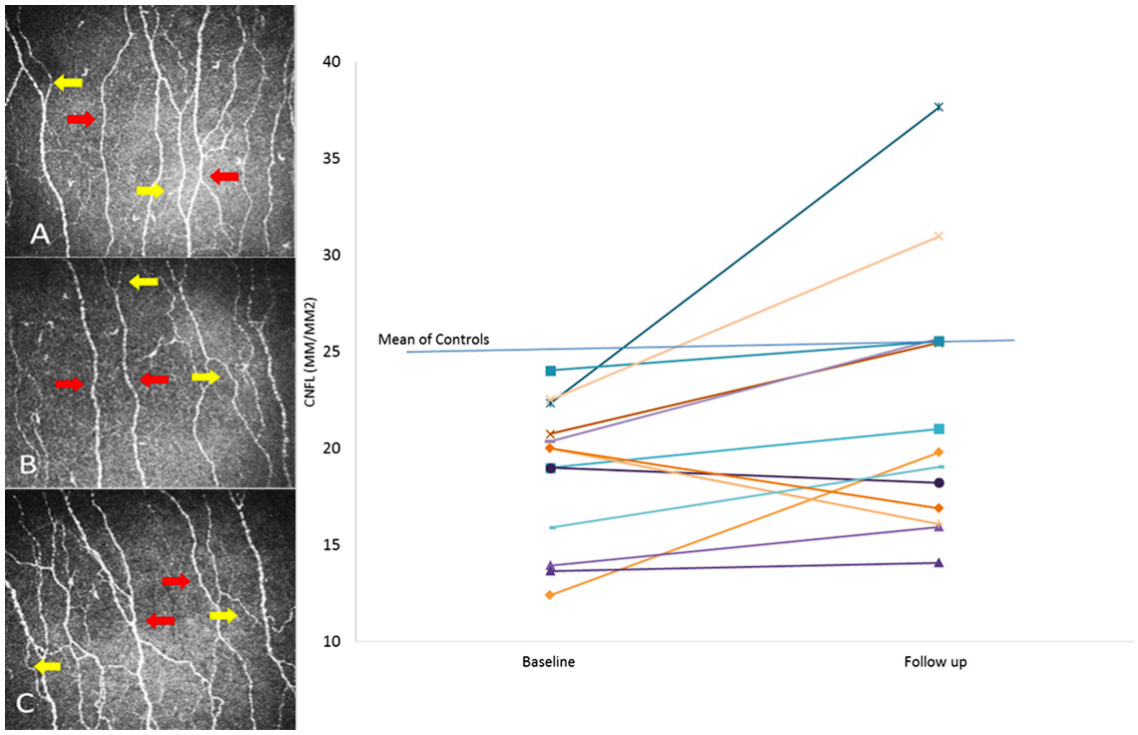
Corneal nerve morphology. CCM image of healthy control (A) and a patient with gastric cancer before (B) and after (C) chemotherapy (Red arrows: main nerve, yellow arrows: branches). (D) Line chart showing consistently reduced CNFL in 13 patients with upper GI cancer at baseline and after the third cycle of chemotherapy (each line represents data from an individual patient).

There was no change in the modified NDS, VPT, CST, CIP, WIP, SNamp, SSNCV, PMNamp, PMNCV or corneal sensation, at follow up (Table 3). However, all CCM parameters increased at follow up and this was significant for CNFL (P = 0.003) (Fig 1). Patients who received Cisplatin or Oxaliplatin showed an increase in CNFL, which was significant in those receiving Oxaliplatin (P = 0.03) (S1 Table). The increase in CNFL showed a significant inverse correlation with the total dose of Cisplatin (r = -0.8, P = 0.05). All patients showed an increase in corneal nerve fibre parameters at follow up except the patients with liver metastases who showed a reduction in CNFD.

**Table 3:** Demographic characteristics and clinical findings of the patients with upper GI cancer before and after chemotherapy.

Parameters	Baseline (13)	Follow up (13)
**NSP**	0.42±0.66	1.91±3.39
**NDS (0–10)**	0.75±1.71	0.17±0.57
**McGill pain index (0–5)**	0.17±0.57	0.45±1.5
**VPT (volts)**	14.04±12.14	15.12±11.62
**CST (**°**C)**	26.42±2.27	24.56±8.04
**WST (**°**C)**	40.00±2.52	40.06±6.71
**CIP (**°**C)**	9.00±6.20	9.40±10.63
**HIP (**°**C)**	47.92±1.78	48.2±1.5
**SSNCV (m/s)**	42.70±7.11	44.6±5.7
**SSNamp (μA)**	9.39±4.41	10.8±4.4
**PMNCV (m/s)**	41.40±3.53	43.7±4.4
**PMNamp (mV)**	3.6±1.95	3.5±2.3
**NCCA (mbars)**	0.53±0.33	0.65±0.57
**CNFD (no./mm** ^**2**^ **)**	26.6±4.8	28.5±7.5
**CNBD (no./mm** ^**2**^ **)**	54.25±25.54	83.7±75.6
**CNFL (mm/mm** ^**2**^ **)**	18.76±3.67	22.0±6.8^#^

All data presented as Mean ± SD

#P<0.05.

NSP (Neuropathy Symptom Profile), NDS (Neuropathy Disability Score), VPT (Vibration perception threshold), CST (Cold Sensation Threshold), WST (Warm Sensation Threshold), CIP (Cold Induced Pain), HIP (Heat Induced Pain), SSNCV (Sural Sensory Nerve Conduction Velocity), SSNamp (Sural Sensory Nerve Amplitude), PMNCV (Peroneal Motor Nerve Conduction Velocity), PMNamp (Peroneal Motor Nerve Amplitude), NCCA (Non-Contact Corneal Aesthesiometer), CNFD (Corneal Nerve Fibre Density), CNBD (Corneal Nerve Branch Density), CNFL (Corneal Nerve Fibre Length), no. (number).

## Discussion

We provide the first report of a detailed study relating CCM to clinical and neurological evaluation in patients with upper GI cancer before and after platinum based chemotherapy. We show no abnormality in a range of established measures of neuropathy including vibration and cold perception as well as cold and heat induced pain thresholds in patients with upper GI cancer. The sural sensory and peroneal motor nerve conduction velocity and warm sensation and heat induced pain threshold demonstrated a mild deficit and an abnormal warm detection threshold has been reported previously in patients with germ cell cancer prior to commencing cisplatin and vinblastine [25]. Neuropathy can be identified clinically in 10–15% and using electrodiagnostic testing in 35–50% of patients with cancer [26]. We now show that CCM detects a marked reduction in corneal nerve morphological parameters in the cohort of patients with upper GI cancer. Furthermore, this reduction was related to the number of abnormal regional lymph nodes and the duration from presentation with symptoms to commencing treatment. Whilst the severity of corneal nerve damage was not related to the histological type or presence and site of metastases, there was an association with the prevalence of metastases and increased mortality, presumably due to more advanced disease.

In the current study, all patients underwent curative-intent or palliative chemotherapy with Cisplatin or Oxaliplatin, which are the standard of care treatments for gastro-esophageal cancers [27]. However, they cause unpredictable neurotoxicity [28] which can be debilitating [1] and refractory to treatment [3, 25]. In the current study Cisplatin and Oxaliplatin containing regimens caused symptomatic neuropathy (CTCAE >1) in 61.5% of patients. The underlying mechanistic basis for these neuropathic symptoms is unclear. Thus Cisplatin treated patients have previously shown no abnormality in cold pain thresholds [29]. Whilst a loss of large fibres has been reported in the sural nerve of 3 patients treated with high dose platinum compounds, unmyelinated axons that actually mediate pain were not assessed [30].

Painful neuropathy has been attributed to regeneration and sprouting of small fibres recently in experimental models of pain [31] and in patients with diabetic neuropathy [32]. Perhaps of relevance, a recent study has shown that paclitaxel-induced neuropathy has a heritable component, driven in part by genes involved in axonal outgrowth [6]. And a recent case report has shown corneal nerve fibre sprouting in a patient with CIPN [21]. Indeed we now show that patients who develop CIPN show an increase in corneal nerve fibre length, which may be consistent with nerve regeneration. These findings are also in line with a previously published study, which showed that intra epidermal nerve fibre density was increased significantly in patients 6 months after chemotherapy [33].

A limitation of this study is the small number of patients attending the follow up visit which may have resulted in bias, but this is due to the high morbidity and mortality from this type of cancer. However, we believe our data are the first to show evidence of a small fibre neuropathy in patients with upper GI cancer, which relates to the burden of disease. The observation that CNFL was increased may provide novel insights into the underlying pathology of CIPN. Further studies assessing a larger group of patients, at an earlier time point and for a longer follow up period are warranted to explore the relevance of an increase in CNFL in relation to nerve regeneration. These data provide further support for the use of CCM as a rapid non-invasive means to image small nerve fibre damage and repair in a range of peripheral neuropathies.

## Supporting Information

S1 Table Demographic characteristics and clinical findings of the patients with upper GI cancer grouped in relation to the type of chemotherapy agent. All data are presented as Mean ± SD. Statistically significant difference compared to baseline visit, *P<0.05.
(PDF)

S2 Table Clinical findings and corneal nerve fibre parameters at baseline and follow chemotherapy in patients showing no difference between patients with (+ve) and without (-ve) symptomatic paraesthesia on the CTCAE criteria. (PDF)

## Abbreviations

CIPN: Chemotherapy induced peripheral neuropathy
CIP: Cold Induced Pain
CST: Cold Sensation Threshold
CTCAE: Common Terminology Criteria of Clinical Adverse Events
CCM: Corneal Confocal Microscopy
CNBD: Corneal nerve branch density
CNFD: Corneal nerve fibre density
CNFL: Corneal nerve fibre length
CNFT: Corneal nerve fibre tortuosity
HbA1c: Haemoglobin A1c
NCS: Nerve conduction studies
NDS: Neuropathy Disability Score
NSP: Neuropathy Symptom Profile
NCCA: Non-Contact Corneal Aesthesiometry
PMNamp: Peroneal motor nerve amplitude
PMNCV: Peroneal motor nerve conduction velocity
SFN: Small fibre neuropathy
SD: Standard Deviation
SSNamp: Sural sensory nerve amplitude
SSNCV: Sural sensory nerve conduction velocity
VPT: Vibration Perception Threshold
WIP: Warm Induced Pain
WST: Warm Sensation Threshold
