# Supplementary material for: Corneal Confocal Microscopy Detects Small Fibre Neuropathy in Patients with Upper Gastrointestinal Cancer and Nerve Regeneration in Chemotherapy Induced Peripheral Neuropathy
Source: PLoS One. 2015 Oct 2;10(10):e0139394. doi: 10.1371/journal.pone.0139394 (PMC4592260; doi:10.1371/journal.pone.0139394)
Supplement: S1 Table — All data are presented as Mean ± SD. Statistically significant difference compared to baseline visit, *P<0.05. (PDF) [file pone.0139394.s001.pdf]

	Cisplatin (n=5)		Oxaliplatin (n=8)	
**Parameters**	Baseline	Follow up	Baseline	Follow up
**Presence of symptoms based on CTCAE (%)**	-	20%(1/5)	-	78%(7/8)*
**NSP**	0.25±0.5	1.00±2.23	0.50±0.75	2.67±4.17
**NDS (0-10)**	0.5±0.57	0.00±0.00	0.88±2.10	0.29±0.75
**McGill pain index (0-5)**	0	0.00±0.00	0.25±0.70	0.83±2.04
**VPT (volts)**	8.7±1.5	12.30±4.85	16.68±14.37	17.14±14.85
**CST (˚C)**	25.75±2.2	25.76±2.44	26.75±2.37	23.71±10.61
**WST (˚C)**	39.00±2.44	37.95±9.36	40.50±2.56	41.57±4.23
**CIP (˚C)**	5.00±3.74	1.16±1.71	11.00±6.39	15.28±10.41
**HIP (˚C)**	47.00±2.70	49.10±1.24	48.38±1.06	47.57±1.39
**SSNCV (m/s)**	44.00±5.65	43.30±3.19	42.38±7.74	46.00±7.61
**SSNamp (µA)**	9.35±0.91	10.76±2.95	9.40±4.98	10.78±5.86
**PMNCV (m/s)**	44.00±4.2	44.92±2.26	40.75±3.32	42.66±5.71
**PMNamp (mV)**	5.00±0.00	3.96±2.80	3.25±2.05	3.16±1.94
**NCCA (mbars)**	0.41±0.36	0.54±0.23	0.62±0.29	0.72±0.73
**CNFD (no./mm^2)**	29.13±4.84	29.09±11.70	25.11±4.42	28.10±4.17
**CNBD (no./mm^2)**	52.50±28.38	105.50±118.53	55.34±25.57	70.10±35.00
**CNFL (mm/mm^2)**	19.50±3.59	23.57±10.20	18.29±3.89	21.07±4.09*
