# Supplementary material for: Corneal Confocal Microscopy Detects Small Fibre Neuropathy in Patients with Upper Gastrointestinal Cancer and Nerve Regeneration in Chemotherapy Induced Peripheral Neuropathy
Source: PLoS One. 2015 Oct 2;10(10):e0139394. doi: 10.1371/journal.pone.0139394 (PMC4592260; doi:10.1371/journal.pone.0139394)
Supplement: S2 Table — (PDF) [file pone.0139394.s002.pdf]

	CTCAE -ve		CTCAE +ve	
**Parameters**	**Baseline**	**Follow up**	**Baseline**	**Follow up**
**VPT (volts)**	17.92±16.32	21.1±16.5	10.88±5.43	10.86±3.9
**CST (˚C)**	26.8±2.5	20.8±11.9	26.8±2.1	27.3±2.1
**WST (˚C)**	39.2±2.2	39.4±10.2	39.8±2.4	40.6±3.5
**CIP (˚C)**	6.7±9.2	1.2±1.7	11.8±7.4	15.3±10.4
**HIP (˚C)**	46.3±3	49.3±1.3	47.2±2.8	47.4±1.1
**SSNCV (m/s)**	37.3±9.3	43.1±3.7	45.8±4.4	45.7±6.9
**SSNamp (µA)**	6.7±2.5	10.9±3.4	10.7±4.3	10.6±5.3
**PMNCV (m/s)**	41.0±5.0	42.7±5.4	43.1±3.6	44.5±3.8
**PMNamp (mV)**	3±2.6	3.2±3	4±2.3	3.8±1.7
**NCCA (mbars)**	0.7±0.4	0.5±0.3	0.7±0.3	0.7±0.7
**CNFD (no./mm^2)**	25.8±5.6	29.9±11.7	25.1±4.6	27.6±3.9
**CNBD (no./mm^2)**	55.1±27.2	120.3±113.4	48.2±29.8	60.9±30.1
**CNFL (mm/mm^2)**	18.3±3.8	25.02±9.8	17.9±3.7	20.2±3.8
